# Cmp7-dependent recruitment of Alx1 to a mitotic nuclear envelope hole in *Schizosaccharomyces pombe*

**DOI:** 10.17912/micropub.biology.002122

**Published:** 2026-04-28

**Authors:** Kaela D. Genereux, Olivia N. Walker, Sage O. Benders, Thanh Truc Le, Nicholas R. Ader

**Affiliations:** 1 Department of Biology, University of North Carolina at Greensboro

## Abstract

The nuclear envelope maintains nucleocytoplasmic compartmentalization. Divergent strategies exist to accommodate the nuclear envelope during mitotic chromatin segregation. While the endosomal sorting complexes required for transport (ESCRT) machinery drives nuclear envelope remodeling during this process, recent work in *Schizosaccharomyces japonicus* suggests an ESCRT-independent pathway promoting nucleocytoplasmic compartmentalization mediated by Alx1 . We investigate if this ESCRT-independent pathway is conserved in *Schizosaccharomyces pombe.* We find Alx1-GFP is recruited to a mitotic nuclear envelope hole in *S. pombe* , but that recruitment depends on the ESCRT machinery. Our data suggest diverse strategies of mitotic nuclear envelope remodeling necessitate varying strategies of promoting nucleocytoplasmic compartmentalization.

**Figure 1. Alx1 is recruited to a mitotic nuclear envelope hole in a Cmp7-dependent manner:** **A:** Live-cell fluorescence micrographs of Alx1-GFP in *S. pombe* representative of cell populations at different stages of the cell cycle. For each subpanel: left, images of Alx1-GFP; middle, sum projection of mCherry-Atb2 (mitotic spindle) and Pcp1-mCherry (SPB); right, magnified views of region of interest (merge at the bottom). Top row shows wild type (WT) cells, and bottom row shows *cmp7∆* cells. Scale bars, 1 µm (main images) and 500 nm (regions of interest). **B** : Percentage of nuclei with Alx1-GFP co-localized with the SPB at the indicated phases of the cell cycle. Data for WT (gray) and *cmp7Δ* (teal) cells are shown. Points and error bars are the mean and range, respectively, from at least three biological replicates; ≥50 nuclei per replicate. Strains used in A and B are listed in Table 1: Alx1-GFP (NASP617; *n* = 268 nuclei); Alx1-GFP, *cmp7∆* (NASP616; *n* = 423 nuclei). **C** : Copy number of Alx1-GFP as a function of time in anaphase B. Black line represents a simple linear regression with the coefficient of determination ( *r* ^2^ ) indicated. Data from three biological replicates are shown, represented by different point shapes (triangle, circle, square) with at least 14 cells per biological replicate. Strains used are listed in Table 1: Alx1-GFP (NASP617; *n* = 106); Fta3-GFP (NASP56). **D** : A model of Alx1 recruitment to the nuclear envelope in *S. pombe* , indicating dependence on Cmp7. Nucleoplasm, blue; cytoplasm, beige; spindle pole body (SPB), dark magenta; mitotic spindle, light magenta.

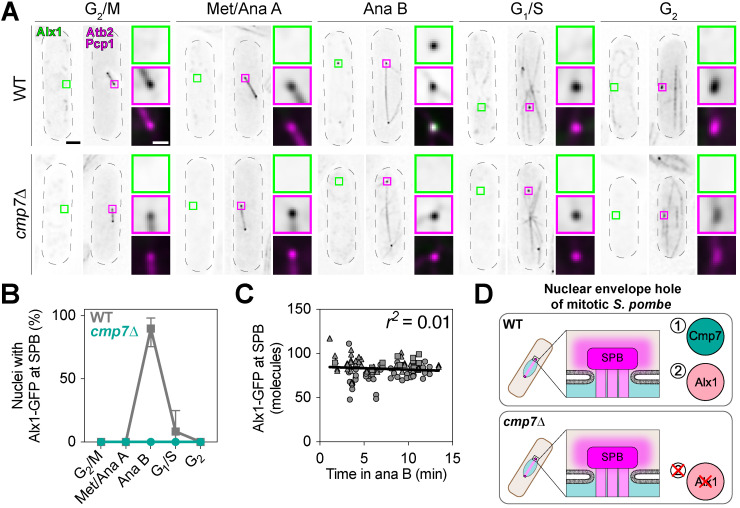



## Description

 The nuclear envelope is critical for maintenance of nucleocytoplasmic compartmentalization. However, nuclear division in many species necessitates a transient disruption of the nuclear envelope to accommodate chromatin segregation by cytoplasmic machinery. At the resolution of nuclear division, holes in the reforming nuclear envelope are sealed by the endosomal sorting complexes required for transport (ESCRT) machinery (Gu et al., 2017; Olmos et al., 2015; Vietri et al., 2015).

The ESCRT machinery remodels membranes across the cell through a group of filament-forming subunits, ESCRT-IIIs, which are canonically targeted to specific membranes by the ESCRT-I/II family of proteins (Burigotto and Carlton, 2025). However, non-canonical recruitment of ESCRT-IIIs by alternative, membrane-specific proteins is also possible. For example, at the nuclear envelope, the unique ESCRT-II/III hybrid protein, Cmp7 , recruits ESCRT-III proteins to facilitate nuclear envelope sealing independent of ESCRT-I/IIs via binding to the integral inner nuclear membrane protein Heh1/Lem2 (LEMD2 in *Homo sapiens;* Gu et al., 2017; Olmos et al., 2016; Thaller et al., 2019; von Appen et al., 2020; Webster et al., 2016).

Recently, the ESCRT-associated protein, Alx1 , has been suggested to play an ESCRT-independent role in the mitotic nuclear envelope sealing of *Schizosaccharomyces japonicus* . While the precise mechanism of this ESCRT-independent pathway remains to be elucidated, it appears to strongly depend on the role of Alx1 (Lee et al., 2020; Sydir et al., 2026). To determine if the presence of an ESCRT-independent nuclear envelope remodeling pathway was conserved across species, we sought to investigate Alx1 involvement in nuclear envelope remodeling in the related fission yeast, *Schizosaccharomyces pombe* . Notably, while closely related to *S. japonicus* , *S. pombe* employ more conservative nuclear envelope remodeling during mitosis *,* simply transiently inserting their microtubule organizing center, the spindle pole body (SPB), into their nuclear envelope to nucleate a mitotic spindle (Ding et al., 1997). Unlike *S. japonicus* , *S. pombe* maintain nucleocytoplasmic compartmentation during mitosis (Arai et al., 2010; Asakawa et al., 2010).

To investigate a potential ESCRT-independent role of Alx1 in mitotic nuclear envelope remodeling in *S. pombe* , we performed live-cell fluorescence microscopy of cells endogenously expressing Alx1-GFP. Unlike core ESCRT-III proteins previously observed by live-cell fluorescence microscopy (Ader et al., 2023), we observed no Alx1-GFP puncta in interphase cells, suggesting that Alx1 is not involved in the formation of intraendosomal vesicles ( Fig. 1A ). Conversely, when we examined mitotic cells, we observed robust Alx1-GFP recruitment to the site of a mitotic nuclear envelope hole, visualized as co-localization of Alx1-GFP and a SPB protein (Pcp1-mCherry) ( Fig. 1A, B). Notably, we did not observe recruitment of Alx1-GFP to a mitotic “tail” of the nuclear envelope as in *S. japonicus* (Lee et al., 2020). Thus, while Alx1 appears to perform a mitotic role at a nuclear envelope hole in *S. pombe* , as in *S. japonicus,* this function appears to be restricted to the mitotic nuclear envelope hole created by the SPB.

While the molecular architecture of in vivo ESCRT assembly remains enigmatic, previous work has sought to determine an absolute number of ESCRT subunits and their stoichiometry at sites of both intraendosomal vesicle formation (Adell et al., 2017) and nuclear envelope constriction/sealing (Ader et al., 2023). Hence, we calculated the absolute number of Alx1-GFP molecules at a nuclear envelope hole throughout mitosis using the kinetochore protein Fta3 as a molecular standard (Ader et al., 2023; Lawrimore et al., 2011). We determined this nuclear envelope hole contains an average of 83 ± 11 molecules of Alx1-GFP and that this level remains relatively constant over the course of anaphase B ( Fig. 1C ). Thus, while the precise role of Alx1 at a nuclear envelope hole remains to be determined, Alx1 appears to be present in an approximate 1:1 stoichiometry with the mitotically recruited ESCRT-III subunit Vps32 (72 molecules; CHMP4 in *Homo sapiens* ; Snf7 in *Saccharomyces cerevisiae* ) and in higher abundance compared to Cmp7 (15 molecules) and Ist1 (30 molecules) (Ader et al., 2023).

We next sought to determine if the presence of Alx1 at a mitotic nuclear envelope hole was consistent with an ESCRT-independent pathway. As all previously observed ESCRT-III recruitment to the nuclear envelope depends on Cmp7 (Ader et al., 2023; Gu et al., 2017; Olmos et al., 2016; Thaller et al., 2019; Vietri et al., 2015), recruitment of Alx1-GFP to a mitotic nuclear envelope hole in cells lacking *cmp7* would suggest an ESCRT-independent role for Alx1 . However, we observed that Alx1-GFP recruitment to a mitotic nuclear envelope hole was completely abolished in *cmp7∆* cells ( Fig. 1A, B). This suggests that in *S. pombe* , unlike *S. japonicus* , the contribution of Alx1 to membrane remodeling of a mitotic nuclear envelope hole is ESCRT-dependent ( Fig. 1D ).

Taken together, our data suggest that even closely related organisms may employ significantly different strategies for nuclear division. While it is tempting to speculate that the presence of an ESCRT-independent pathway for post-mitotic nuclear envelope sealing in *S. japonicus* may be a consequence of this yeast's more “open” mitosis compared to *S. pombe* , further work across more diverse species is needed.

## Methods

***S. pombe culture and strain construction.*** *S. pombe* strains were cultured as standard (Moreno et al., 1991), with all experiments performed in either agar plates or liquid culture of yeast extract supplemented with 250 mg/L adenine, histidine, leucine, uracil, and lysine hydrochloride (YE5S). The pFA6a-MX6-based drug-resistance cassettes were used for carboxy (C)-terminal tagging (Bähler et al., 1998; Hentges et al., 2005). A plasmid Editor (ApE) 2.0.61 was used for visualization and virtual manipulation of DNA sequences (Davis and Jorgensen, 2022), with *S. pombe* genome information accessed through PomBase (Carme et al., 2026). PCR of whole yeast (colony PCR) (Huxley et al., 1990) was used to confirm genomic integration following lithium acetate transformation (Murray et al., 2016). Monomeric enhanced GFP (mEGFP) used for strain creation throughoutwith a 3×HA linker; abbreviated as GFP in Figure and Description.

***Live-cell fluorescence microscopy.*** Cells expressing fluorescently tagged proteins were cultured to logarithmic phase (OD _600_ 0.4–0.8), concentrated by brief centrifugation, and mounted on 1.4% agarose in Edinburgh minimal medium supplemented with 250 mg/L adenine, histidine, leucine, uracil, and lysine hydrochloride (EMM5S) pad sandwiched with a no. 1.5 glass coverslip, a glass slide, and then sealed with VALAP (1:1:1, vaseline:lanonin:paraffin) for imaging. Live-cell fluorescence microscopy was performed using a Nikon microscope imaging system with an incubated stage (Oko Lab) set to 32˚C. An LED light source (Nikon, D-LEDI) was used for illumination. For GFP: 475 nm excitation with a FITC filter cube (excitation 465–495 nm, emission 515–555 nm, long pass 505 nm). For mCherry: 550 nm excitation with an mCherry filter set (excitation 540–580, emission 593–667 nm, long pass 585 nm). Channels were acquired sequentially on an ORCA-Fusion BT Digital CMOS camera (Hamamatsu C15440-20UP) using a Plan Apo Lambda D 100× oil objective with a numerical aperture of 1.45. Z-stacks spanning the nuclear envelope region (~3.5–4.8 µm) were acquired every 200 nm to visualize Alx1-GFP localization at the mitotic nuclear envelope. Widefield images were deconvolved in NIS-Elements using a Richardson-Lucy method. Cells were classified into phases as previously published using mCherry- Atb2 (Ader et al., 2023). All Alx1-GFP images are shown at equivalent display range, set to maximize display range for Alx1-GFP image shown in WT anaphase B ( Fig. 1A ). For images in the mCherry channel (mCherry- Atb2 and Pcp1-mCherry), the gamma has been adjusted to 0.05 to allow both fluorophores to be displayed without pixel saturation. mCherry images were manually adjusted to to maximize display range for each image. Figures were designed and prepared using Prism, Adobe Illustrator 2026, and Fiji.

***Quantification of the average copy number of Alx1 subunits.*** Cells were prepared for imaging by overnight culture as described in live-cell fluorescence microscopy. Cells (NASP56) expressing Fta3-GFP were mounted on the same pad as Alx1-GFP cells and imaged as above. Raw images were analyzed using a custom MATLAB script, ‘Find_n_Fit_Spots' (https://github.com/LusKingLab/Find_n_Fit_Spots_3D) as previously described (Ader et al., 2023).

## Reagents

**Table:** 

**Table 1.** List of *S. pombe* strains used in this study.		
**Strain (NASP)**	Genotype	Origin
56	fta3 ::fta3-mEGFP:kanMX6 | leu1-32 | ura4-D18 | ade6-M210 | h+	Ader et al., 2023
616	alx1 ::alx1-3xHA-mEGFP:kanMX6 | cmp7 ::hygMX6 |&nbsp; pcp1 ::pcp1-mCherry:natMX6 | aurR-pnda3-mCherry- atb2 | leu1-32 | ura4-D18	This work&nbsp;
617	alx1 ::alx1-3xHA-mEGFP:kanMX6 | pcp1 ::pcp1-mCherry:natMX6 | aurR-pnda3-mCherry- atb2 | leu1-32 | ura4-D18	This work

**Table:** 

**Table 2.** List of primers used in this study.	
Primer Name&nbsp;	Sequence
prNA253_ alx1 _L3	GCTTTAGAGAAGCTGTTGATGCCAAG&nbsp;
prNA254_ alx1 _L4	ttaattaacccggggatccgTTTTTTTGACGATTTAAACTTGATTTTATGGATTTCAGG
prNA255_ alx1 _L5	gtttaaacgagctcgaattcGATTATCATATGTGATCATATAGTATTAAATCCGCATACA
prNA256_ alx1 _L6	AAATGCTTCAACGGTGTGGATGG&nbsp;

**Table:** 

Table 3. Plasmids used in this study.&nbsp;			
**Identifier&nbsp;**	**Name/Description**	**Usage&nbsp;**	**Origin&nbsp;**
pNA8&nbsp;	pNA8_pFA6a-3xHA-mEGFP-KanMX6	Template for PCR based chromosomal integration of 3×HA-mEGFP ORF	Ader et al., 2023
